# Retraction: GREB1 Functions as a Growth Promoter and Is Modulated by IL6/STAT3 in Breast Cancer

**DOI:** 10.1371/journal.pone.0102287

**Published:** 2014-07-15

**Authors:** The PLOS ONE Editors

Following the publication of this article, Dr. Marc Lippman from the University of Miami Miller School of Medicine raised concerns that the article was based on work completed in his laboratory and which had been employed by the authors without his knowledge or permission.

Dr. Lippman's concerns were evaluated by a review panel at Morehouse School of Medicine which concluded that there was substantial evidence in support of Dr. Lippman's claims and advised that the publication should be retracted. The concerns have also been raised to the attention of the University of Miami which advised proceeding as recommended by Morehouse School of Medicine.

In the light of the recommendation of Morehouse School of Medicine, the editors retract this publication.

We also wish to make readers aware that references 16 and 21 in the original paper were cited with incorrect author details. The two references relate to the same article and the correct full citation is as below:

Correlation of GREB1 mRNA with protein expression in breast cancer: validation of a novel GREB1 monoclonal antibody. Breast Cancer Res Treat. 2010 Jul;122(2):371-80. doi: 10.1007/s10549-009-0584-x. Hnatyszyn HJ, Liu M, Hilger A, Herbert L, Gomez-Fernandez CR, Jorda M, Thomas D, Rae JM, El-Ashry D, Lippman ME.
